# Correlation Between Serum Tumor Necrosis Factor-Alpha (TNF-α) and Clinical Severity of Tuberculosis: A Hospital-Based Study

**DOI:** 10.7759/cureus.35626

**Published:** 2023-02-28

**Authors:** Touseef Yaqoob Beig, Umar H Khan, Basharat Ahmed Ganie, Sheikh Tahir, Sanaullah Shah, Gh Nabi Dhobi

**Affiliations:** 1 Internal Medicine, Sher-i-Kashmir Institute of Medical Sciences, Srinagar, IND; 2 Geriatrics, Sher-i-Kashmir Institute of Medical Sciences, Srinagar, IND; 3 Pulmonary Medicine, Sher-i-Kashmir Institute of Medical Sciences, Srinagar, IND

**Keywords:** tnf-α, controls, cases, clinical severity, cytokines, tuberculosis

## Abstract

Aim: The main aim of this study was to assess the correlation between serum tumor necrosis factor-alpha (TNF-α) levels the and clinical severity of tuberculosis.

Methods: This was a hospital-based case-control prospective study and was conducted at the Sher-i-Kashmir Institute of Medical Sciences, a tertiary care hospital in the northern part of India, from May 2016 to May 2018. The subjects were recruited in the study considering inclusion and exclusion criteria. All patients with pulmonary tuberculosis as well as patients with extrapulmonary tuberculosis were included as subjects and a clinical severity score based on anemia, weight loss, presence of hypoxia, and radiological features was calculated and compared with TNF-α levels. Age- and sex-matched healthy individuals were recruited as controls.

Results: A total of 75 subjects comprising 50 cases and 25 controls were taken for the study. There were 34 (68.0%) patients with elevated TNF-α levels while only 16 (32.0%) patients had normal TNF-α levels. And, TNF-α levels were normal in 21 (84%) control subjects as compared to tuberculosis (TB) patients. Such difference in serum TNF-α levels between cases and controls was statistically significant (p<0.05). The mean serum TNF-α level in TB cases was 1265.63 pg/mL, while the mean serum TNF-α level in controls was 312.06 pg/mL. The difference in serum TNF-α levels between the two groups was statistically significant (p<0.01). We observed a significant increase in serum TNF-α levels with the increase in clinical severity score.

Conclusion: Serum TNF-α levels were significantly associated with increased severity of TB.

## Introduction

Tuberculosis (TB) is a major cause of death around the world. The infected cells and tissue macrophages in tuberculosis infection release a significant amount of cytokines. Studies have indicated the key role of TNF-α in infection control, mycobacteria eradication, and the development of granulomas [1]. In HIV-TB co-infected individuals, T cells produce less TNF-α, which may account for the individuals’ poor granuloma development [2]. Studies have demonstrated a clear correlation between blood levels of tumor necrosis factor-alpha (TNF-α) and interleukin-1 (IL-1) and the severity of tissue infection, as well as a correlation between fever production and other consumptive signs of the illness [3,4].

The cell-mediated immune response is essential for TB resistance, mainly through the production of cytokines, such as interleukin-2 (IL-2), interferon-gamma (IFN-γ), interleukin-12 (IL-12), interleukin-18 (IL-18) and tumor necrosis factor-alpha (TNF-α). When glycolipid antigen molecules are delivered to T lymphocyte cells by major histocompatibility complex (MHC) class I proteins in conjunction with cluster of differentiation 1d (CD1d) glycoprotein molecules, the TCD8^+^ cells (CTLs {cytotoxic lymphocytes}) engage in cytotoxic actions [5]. In tuberculosis, CTL cells might have a protective role through a variety of mechanisms - (1) IFN-γ and TNF-α are produced; (2) *Mycobacterium tuberculosis *antigens presented by MHC coupled to HLA-I (human leucocyte antigen-I) complex are recognized; and (3) infected cells are induced to undergo apoptosis [6,7]. Because of the multiple actions of IL-12, IFN-γ, IL-4, and IL-10, the role of cytokines produced by Th1/Th2 cells is complicated (pro-inflammatory and anti-inflammatory cytokines). Recent studies investigated the facets of this complicated interaction, including the suppression of interferon-gamma by interleukin-10 (IL-10) [8], the stimulation of IFN-γ and IL-10 production by IL-12 [9], the stimulation of IFN-γ and IL-10 production by TCD4^+^ cells in the bronchoalveolar lavage (BAL) fluid of patients with pulmonary TB [10], and the association of Th2 cytokines with histopathologic changes in patients with TB [11]. Furthermore, there is a physiopathologic link between serum TNF-α and the evolution of mycobacteria infection, according to several research studies. In mice that had been exposed to *M. tuberculosis *six months earlier, TNF-α neutralization increased the lung bacterial load [12]. Both the synthesis of TNF-α and the virulence of *M. tuberculosis *in human monocytes, as well as the rise in serum TNF-α levels and the worsening of a patient's clinical condition in cases of severe TB, are related [13,14]. Interleukin-1beta (IL-1β), interleukin-4 (IL-4), interferon-gamma (IFN-γ), transforming growth factor beta (TGF-β) released by tuberculosis patients' peripheral blood mononuclear cells in in vitro research studies as well as the research that examined the amounts of TNF-α in BAL fluid demonstrated that more the lung involvement present, higher the cytokine production [15,16]. Therefore, the objective of this study was to correlate serum TNF-α levels with the severity of TB.

## Materials and methods

This is a prospective study conducted at the Sher-i-Kashmir Institute of Medical Sciences in the Department of Internal Medicine from May 2016 to May 2018. The informed consent of all patients and healthy controls were obtained. This study investigated the relationship between serum TNF-α levels and the clinical severity of tuberculosis. Clinical data, including anemia, fever, weight loss, dyspnea, night sweats, cough, sputum, and systemic examination were analyzed for patients. Diagnostic tests, including purified protein derivative (PPD) skin testing, erythrocyte sedimentation rate and blood counts, thoracic x-rays, abdominal x-rays, CT/MRI brain, CT scans of the chest, and CT scans of the abdomen/CT enterography, were also performed, depending on the location of the disease. Acid-fast bacilli positivity in fluid, tissue, or culture in each case, or a high adenosine deaminase (ADA) (in CSF, pleural and ascitic fluid), were used to diagnose tuberculosis.

The results of the thoracic radiography, the computed tomographic (CT) scan of the chest, CT brain, MRI (magnetic resonance imaging) brain, CT scan abdomen, and PPD skin test were coded as 0 (negative) or 1 (positive). The clinical severity of each case was determined through the following score: without anemia=0, with mild anemia=1 (11 g/dL or more), with moderate anemia=2 (8-11 g/dL), with severe anemia=3 (≤8 g/dL); without weight loss=0, with weight loss=1; without hypoxia=0, with hypoxia=1; thoracic x-ray/CT chest normal=0, thoracic x-ray/CT chest altered without cavity=1, thoracic x-ray/CT chest altered with cavity=2.

TNF-α assay

Sera were obtained from diagnosed pulmonary and extrapulmonary patients, and healthy individuals (controls). A highly sensitive diaclone enzyme-linked immunosorbent assay (ELISA) method was used for measuring TNF-α levels. The sensitivity or the minimum detectable dose of TNF-α using this high-sensitivity diaclone TNF-α ELISA kit was found to be 0.5 pg/mL. This was determined by adding three standard deviations to the mean OD obtained when the zero standards were assayed 35 times. However, we considered 1.0 pg/mL as the minimum value below which the interleukin levels were treated as undetectable in order to be in line with most of the moderately sensitive ELISA kits used in our state (northern part of India).

Statistical analysis

IBM SPSS Statistics version 27.0 (released 2020; Armonk, NY: IBM Corp.) for Windows was used for the statistical analysis. Continuous data were presented as descriptive statistics (i.e., mean, standard deviation, range), whereas categorical variables were represented by frequencies and percentages. Also, the association was done by using the chi-square test and r-value calculation using Pearson's correlation, the correlations between each case's TNF-α level and all clinical and diagnostic variables, and all results were discussed at 5% level of significance, i.e., p<0.05.

Ethics

The study was carried out in accordance with the institutional ethical committee's recommendations and the 2013 revision of the 1964 Helsinki Declaration. The Institutional Ethics Committee (IEC) of Sher-i-Kashmir Institute of Medical Sciences (SKIMS) gave its approval to this study.

## Results

Seventy-five (n=75) subjects including 50 cases and 25 controls were recruited for the study. Almost all the cases attended the hospital with a clinical presentation of TB. In this study, 54% (n=27) of the cases were males and 46% (n=23) were females with male to female ratio of 1:0.85. Based on age, the patients were grouped into two categories, less than 40 years and greater than 40 years of age. The number of cases in the age group of >40 years (n=30; 60%) exceeded those <40 years (n=20; 40%). The mean age of the patients was 43.5±13.9 years. Based on the smoking status, 20 (40%) patients were smokers, 16 (32%) were non-smokers and 14 (28%) were passive smokers. The differences in smoking among cases and controls were statistically significant (p<0.05). The subjects were considered non-smokers only if up to the day of sample collection they had not used tobacco and current smokers if they are smoking presently or had quit smoking in the last <6 months before sample collection. There was almost a similar representation of cases from rural and urban areas. There were overall no significant differences between cases and controls among different parameters like weight, socioeconomic status, and TB-associated co-morbidities (p>0.05). However, positive history of TB among subjects was significantly associated with cases (p<0.05) (Table 1).

**Table 1:** Demographic and risk factors in TB cases and controls of the study. PFT: pulmonary function test, TNF-α: tumor necrosis factor-alpha

Variables	Categories	Cases		Controls		p-Value
		n=50	%	n=25	%	
Age	<40	20	40	11	44	>0.05
	>40	30	60	14	56	
Gender	Male	27	54	15	60	>0.05
	Female	23	46	10	40	
Dwelling	Urban	26	52	17	68	>0.05
	Rural	24	48	8	32	
Smoking status	Smokers	20	40	8	32	<0.05
	Non-smokers	16	32	8	32	
	Passive smokers	14	28	09	36	
Weight (kg)	<70	35	70	18	72	>0.05
	>70	15	30	7	28	
PFT	Normal	26	52	0	0	NA
	Abnormal	24	48	0	0	
Socio economic status	Good	30	60	15	60	>0.05
	Average	15	30	7	28	
	Poor	5	10	3	12	
Family history	Yes	26	52	3	12	<0.05
	No	24	48	22	88	
Co-morbidities	Diabetes	5	10	5	20	>0.05
	Hypertension	4	8	8	32	
	Diabetes + hypertension	3	6	6	24	
	Nil	38	76	6	24	
TNF-α	Normal	16	32	21	84	<0.05
	High	34	68	4	16	

There were 34 (68%) patients with elevated TNF-α levels while only 16 (32%) patients had normal TNF-α levels, the difference was statistically significant (p<0.05) (Table 1). A total of 25 consecutive healthy controls were recruited to elucidate the various clinic pathologic characteristics listed in Table 1. In this study, the total number of male controls was 15 (60%) while that of females was 10 (40%). The number of control subjects in the age group of <40 years (n=11; 44%) was relatively lower than >40 years (n=14; 56%). The mean age of the control subjects was 39.45±12.2 years. Based on the smoking status eight (32%) controls were smokers, eight (32%) were non-smokers, and nine (36%) were passive smokers. Like cases, controls were similar to cases in their weights, socioeconomic statuses, and places of residence.

**Figure 1:**
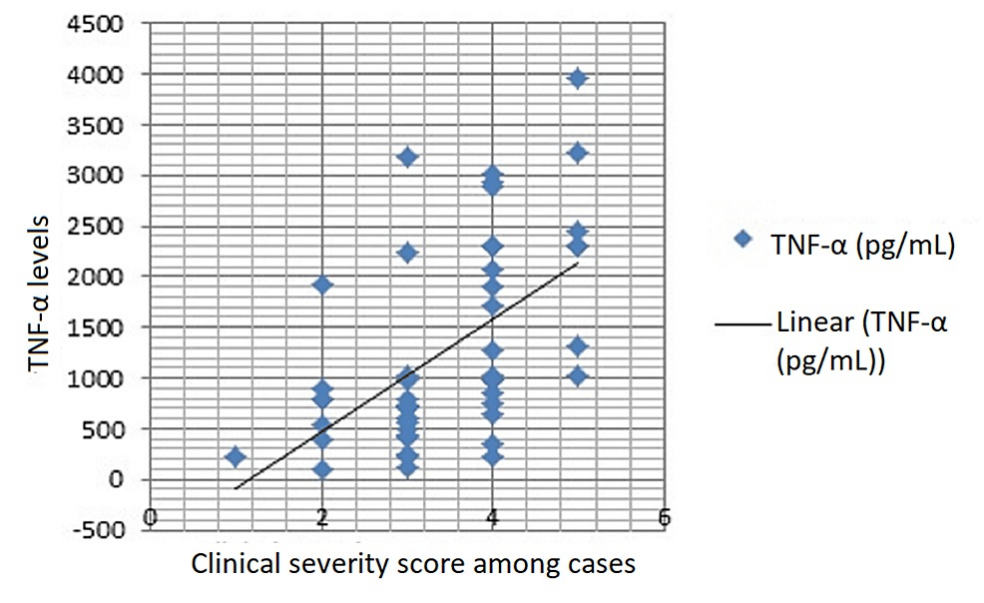
Correlation between serum TNF-α levels and clinical severity among cases. TNF-α: tumor necrosis factor-alpha

There were 34 (68%) patients with elevated TNF-α levels while only 16 (32%) patients had normal TNF-α levels. While TNF-α levels were majorly normal in control subjects 21 (84%) as compared to TB cases. Such differences in serum TNF-α levels between cases and controls were statistically significant (p<0.05) (Table 1). In TB cases, the mean of serum TNF-α levels was 1265.33 pg/mL, while as the mean serum TNF-α levels in controls was 312.06 pg/mL, the difference in serum TNF-α levels between the two groups was statistically significant (p<0.001) (Table 1). The correlation between serum TNF-α levels and clinical severity in cases is depicted in Figure 1.

The association of TNF-α serum levels and demographic variables was analyzed, and it was found that there was no significant difference (p>0.05) among cases and controls with respect to all the subgroups of age, gender, place of residence, socioeconomic status, and other associated co-morbidities. The presence of smoking among cases and controls was significant with elevated serum TNF-α levels (p<0.05). Similarly, a history of TB among blood relatives in combination with increased TNF-α serum levels showed a significant association with TB (Table 2).

**Table 2:** Correlation between TNF-α levels and smoking and positive history of TB among blood relatives in relation to TB development. TNF-α: tumor necrosis factor-alpha; TB: tuberculosis

Smoking status	Cases	Controls	OR (95% CI)
Non-smokers + TNF-α normal	12	10	Reference
Non-smokers + TNF-α high	10	2	0.8 (1.23-29.21)
Smokers + TNF-α normal	4	11	0.24 (3.91-39.12)
Smokers + TNF-α high	24	12	3.45 (2.22-19.31)
Family history			
No history of TB + TNF-α normal	14	1	Reference
No history of TB + TNF-α high	10	2	1.89 (1.23-40.21)
History of TB + TNF-α normal	10	8	0.89 (0.71-36.21)
History of TB + TNF-α high	16	3	4.45 (4.28-49.91)

## Discussion

In the current study, a total of 50 TB patients were considered for analysis with varied clinical manifestations of TB. We attempted to demonstrate that TNF-α could potentially act as a cytotoxic agent, especially when increased blood levels were observed. Cytokine escape into the serum is the cause of TNF-α being present in peripheral blood circulation. *M. tuberculosis* stimulation of tissues and inflammatory cells activates monocyte-macrophages to generate this cytokine [17].

The details of the patient groups displayed, highlight that TNF-α plays a key role in the various clinical manifestations of TB. The control group and the TB group had significantly different serum TNF-α levels. We found that the clinical severity of the disease and serum TNF-α levels were positively correlated when the cases were categorized as per clinical severity scores [18,19]. The results of the subgroup analysis were also intriguing. Severe lung lesions with enhanced tissue damage were found in multi-drug resistant (MDR)-TB subjects. The serum levels of TNF-α in the MDR-TB group have been shown to increase from very low to very high.

However, in some cases, serum TNF-α levels were very low. We hypothesize that the immunological response to the infectious pathogen may have been quite subpar in these patients. Although some individuals had severe lymphadenopathy and necrosis that could be seen using imaging techniques, some of them had normal serum TNF-α values. In some patients with lymph node TB, we hypothesize that the immune response may be compartmentalized. The extent of pulmonary tissue damage was higher in those with the highest serum TNF-α levels. We speculate that TNF-α may directly contribute to this behavior.

We measured blood TNF-α levels sequentially in a small number of patients and found that, with a decrease in serum TNF-α levels, the patient's physical condition invariably improved. On the other hand, a poor clinical course was observed when serum TNF-α levels did not drop throughout the evolution. These findings suggest that the serum TNF-α levels may be a reliable indicator of the clinical course of TB patients. After demonstrating that declines in sputum IFN-γ, TNF-α, and IL-8 closely follow and even precede mycobacterial clearance, Ribeiro-Rodrigues et al. concluded that these cytokines may be reliable markers of disease activity and inflammation in TB [20].

Additionally, we observed a positive association between serum TNF-α levels and weight loss, abnormal chest x-rays, and positive PPD skin tests; similar findings were observed by Andrade et al. [19]. Other studies also noted this relationship between serum TNF-α levels and weight loss [18]. When comparing these levels in patients with cancer and low weight loss, Bossola et al. found that individuals with cancer and severe weight loss had considerably greater serum TNF-α concentrations [21]. Leptin and serum TNF-α levels were hypothesized by Cakir et al. to play a key role in weight loss in pulmonary TB patients [22]. Another study found a link between weight loss in rats and prolonged TNF-α injection [23].

We observed that those with abnormal chest x-rays had higher serum TNF-α levels, Andrade et al. reported comparable findings [19]. Tsao et al. demonstrated that patients with big cavities had considerably greater levels of TNF-α, IL-1β, and IL-6 in their bronchoalveolar lavage fluid than those without cavities or with smaller cavities [24,25]. The relative excess of TNF-α and IL-1β, associated with soluble TNF-α receptor secretion imbalance, was also demonstrated by these authors to be related to the pulmonary cavities in individuals with active tuberculosis (TB) [24]. The association between serum TNF-α levels and abnormal x-rays was, however, the subject of very few investigations in the medical literature, and more research is required to fully comprehend these phenomena. Additionally, we discovered that in patients who had a positive PPD skin test, the serum TNF-α level was increased. According to Chu et al., the amount of cell staining for TNF-α and IL-1 persisted at high levels in the skin of six healthy individuals during the PPD-induced reaction [25,26]. According to the authors, TNF-α may be crucial in the emergence of delayed-type hypersensitivity in humans. As a result, we added the fact that patients with positive PPD skin tests often have high serum TNF-α levels, which may indicate a link between the cytokine's serum level and its tissue activity.

Patients who had smoked in various ways demonstrated a substantial link with TB, and the study's significant finding was the presence of TB in blood relatives. Smoke is one of the allergens that has been found to alter IgE repertoires in the nasal mucosa. Additionally, the stimulation of the allergen in the mucosa may cause a sequential switch from IgG or IgA to IgE. Also, it has been found that mucosal locations produce almost all of the serum IgE, and nasal mucosa IgE-secreting B cell and plasma cell populations see a considerably larger rise than peripheral cell populations. Although there are data on the heritability of TB, the specific mechanism of genetic regulation has remained elusive [6,27]. Numerous studies have discovered a strong correlation between first-degree relatives and TB cases. Genetic and environmental exposure, either separately or together, could be the plausible cause of a higher prevalence of TB in blood relatives.

One of the objectives of our study was to demonstrate the relationship between high serum TNF-α levels and extremely severe clinical TB with considerable tissue damage. On the other hand, we saw that patient clinical improvement was accompanied by a decline in the serum cytokine level. For instance, when the cytokine serum level is increased for an extended period, we hypothesize that lowering high TNF-α serum levels may reduce tissue damage.

Limitation

The study's limitation is the paucity of knowledge that exists currently about TNF-α and TB in our group. However, selection or recall bias and the relatively smaller sample size could be the study's weak points. This bias is reduced by using the same hospital setting.

## Conclusions

Future standard TB clinical evaluations should incorporate TNF-α periodic measures using bioassay or ELISA technologies as they may help in understanding the consequences of tuberculosis. More research, nevertheless, is required before drawing firm conclusions. Today, *M. tuberculosis *elimination is the main focus of TB treatment. We recommend that TNF-α immune modulation be examined in *M. tuberculosis *therapy in future investigations, particularly in instances with extremely high TNF-α serum levels.
